# Further investigation of confirmed urinary tract infection (UTI) in children under five years: a systematic review

**DOI:** 10.1186/1471-2431-5-2

**Published:** 2005-03-15

**Authors:** Marie E Westwood, Penny F Whiting, Julie Cooper, Ian S Watt, Jos Kleijnen

**Affiliations:** 1 Centre for Reviews and Dissemination, University of York, England; 2 MRC Health Services Research Collaboration, University of Bristol, England; 3 Department of Radiology, York District Hospital, York, England; 4 Department of Health Sciences, University of York, England

## Abstract

**Background:**

Further investigation of confirmed UTI in children aims to prevent renal scarring and future complications.

**Methods:**

We conducted a systematic review to determine the most effective approach to the further investigation of confirmed urinary tract infection (UTI) in children under five years of age.

**Results:**

73 studies were included. Many studies had methodological limitations or were poorly reported.

*Effectiveness of further investigations: *One study found that routine imaging did not lead to a reduction in recurrent UTIs or renal scarring.

*Diagnostic accuracy: *The studies do not support the use of less invasive tests such as ultrasound as an alternative to renal scintigraphy, either to rule out infection of the upper urinary tract (LR- = 0.57, 95%CI: 0.47, 0.68) and thus to exclude patients from further investigation or to detect renal scarring (LR+ = 3.5, 95% CI: 2.5, 4.8). None of the tests investigated can accurately predict the development of renal scarring. The available evidence supports the consideration of contrast-enhanced ultrasound techniques for detecting vesico-ureteric reflux (VUR), as an alternative to micturating cystourethrography (MCUG) (LR+ = 14.1, 95% CI: 9.5, 20.8; LR- = 0.20, 95%CI: 0.13, 0.29); these techniques have the advantage of not requiring exposure to ionising radiation.

**Conclusion:**

There is no evidence to support the clinical effectiveness of routine investigation of children with confirmed UTI. Primary research on the effectiveness, in terms of improved patient outcome, of testing at all stages in the investigation of confirmed urinary tract infection is urgently required.

## Background

UTI in children is an important clinical problem. Renal scarring, which occurs in a small proportion of children (approximately 6%[1]), is the most important outcome of infection as it is associated with significant future complications[2], and ultimately with end stage renal disease[3]. Young children are considered particularly vulnerable to renal scarring and its consequences[4]. However, a recently completed 20-year follow-up study suggested that compensatory mechanisms mean no significant changes in overall GFR occur in patients with unilateral scaring[1], and the risk of hypertension is low in all patients (regardless of the degree of scarring)[5].

Current UK recommendations state that all children under 5 years of age should be investigated after their first confirmed UTI[6,7], although the benefit from this strategy has been questioned[8].

Further investigation of children with confirmed UTI has a number of different clinical aims: the localisation of infection, the prediction and detection of renal scarring and the detection of VUR. The current reference standards for these investigations are Tc-99 m-DMSA renal scintigraphy (DMSA scan) for the localisation of infection and for the detection and prediction of renal scarring, and micturating cystourethrography (MCUG) for the detection of VUR. These investigations have the disadvantages of being invasive and involving exposure to ionising radiation. It is desirable to minimise the number of invasive examinations and radiation load to which children are exposed. Alternative tests that offer a potential advantage over the current reference standards, such as ultrasound or laboratory-based tests, are therefore required. An additional aim of the investigation of children with UTI is the detection of anatomical abnormalities that may be amenable to surgery, and a role has been suggested for ultrasound in this context. We did not identify any studies evaluating tests with this objective; therefore it could not be assessed in this review. However, a recently published observational study has suggested that routine ultrasound post-UTI, in children under five years, does not change management[9]. The role of pre-natal ultrasound is unclear[9,10] and was outside the scope of this review.

We reviewed the diagnostic accuracy of tests evaluated for the further investigation of UTI together with evidence of their long-term effectiveness, with a view to determining the optimum diagnostic pathway. A previous systematic review has evaluated ultrasound for the detection of scarring[11]. This review was published in 1999 with searches undertaken in 1997 and only included 10 studies. We are unaware of any other systematic reviews in this area. This review therefore represents the most complete review of the area.

## Methods

We assembled a database of published and unpublished literature from systematic searches of 16 electronic databases (inception to between October 2002 and February 2003), hand searching of 12 journals, consultation with experts in the field, and screening bibliographies of included studies. Update searches were conducted in May 2004. There were no language restrictions. Full details of the search strategy will be reported elsewhere[12].

We included controlled trials that compared different imaging strategies and reported patient based outcomes. We also included diagnostic cohort studies that included at least 20 children, some of whom were aged five years or under, and that reported sufficient information to construct 2 × 2 tables. Table 1 presents a summary of the tests evaluated in this review and details the potential advantages that these tests have over the current reference standards. Although other tests have been evaluated, this article will focus on those offering potential advantages over the current reference standard. Studies had to compare one of the index tests listed in this table to the listed reference standard, for each of the different aims.

**Table 1:** Summary of tests used for different clinical applications

**Aim**	**Current Reference standard**	**Tests**	**Advantage over the reference standard**
Localisation of infection	Tc-99 m-DMSA renal scintigraphy	Clinical features Laboratory-based tests Ultrasound	All less invasive, no exposure to ionising radiation
Detection of reflux	Micturating cystourethrography (MCUG)	Ultrasound	Less invasive, no exposure to ionising radiation
		Indirect radionuclide cystography	Single procedure test for reflux and scarring
Prediction of renal scarring	Follow-up Tc-99 m-DMSA renal scintigraphy	Clinical features	Would allow earlier prediction of children at risk of renal scarring
		Ultrasound	
		MCUG	
		Acute DMSA renal scintigraphy	
Detection of renal scarring	Tc-99 m-DMSA renal scintigraphy	Ultrasound	Less invasive, no exposure to ionising radiation
		Intravenous pyelography (IVP)	Provides a detailed anatomic map, considered essential where surgery is planned.
		Radionuclide cystography	Advocated as a single procedure test for reflux and scarring

Two reviewers independently screened titles and abstracts for relevance, any disagreements were resolved by consensus. One reviewer performed inclusion assessment, data extraction and quality assessment; a second reviewer checked this. We extracted 2 × 2 data from each of the studies and used this to calculate estimates of test performance. Where insufficient details were reported, we contacted authors to request further information. We assessed the methodological quality of diagnostic accuracy studies using QUADAS[13]. Individual QUADAS items were used to investigate heterogeneity and to present a detailed assessment of quality to the reader.

We analysed results grouped by clinical aim. Where studies presented more than one estimate of test performance for the same test, we only included one estimate in the pooled analysis. We aimed to select the most appropriate data set or the one most similar to that used by other studies in terms of population, technique or unit of analysis. For example, data for tests used to localise infection were analysed by patient in preference to kidney or renal unit (kidney and ureter), whereas data for tests used to detect VUR were analysed by renal unit. For each test, or test combination, we calculated the range in sensitivity, specificity, positive (LR+) and negative (LR-) likelihood ratios, and diagnostic odds ratios (DOR). Where sufficient studies were available, pooled likelihood ratios were calculated for each test[14]. Heterogeneity of likelihood ratios was investigated using the Q statistic[15] and through visual examination of forest plots of study results[16]. We presented individual studies results graphically by plotting estimates of sensitivity and specificity in receiver operating characteristic (ROC) space. Where sufficient data were available, we used regression analysis to investigate heterogeneity. We extended the summary ROC (sROC) model[17], estimated by regressing D (log DOR) against S (logit true positive rate – logit false positive rate), weighted according to sample size, to include the variables for patient age (<2 years, <5 years, <12 years and <18 years), geographic region, and QUADAS items[18]. For ultrasound for the detection of VUR a variable for ultrasound technique (contrast-enhanced or standard) was also included.

## Results

**Figure 1:**
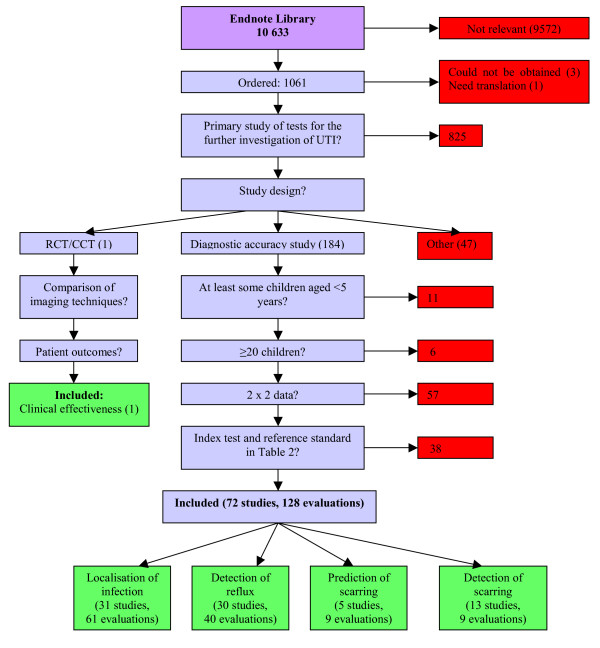
Flow of studies through the review

We identified more than 10,000 studies. Of these, 73 studies met our inclusion criteria: 72 diagnostic accuracy studies and one RCT of the clinical effectiveness of investigation. Figure 1 shows the flow of studies through the review process. The results of individual included studies are presented [see Additional file 1]. Table 2 presents summary results for each included test.

**Table 2:** Summary of the results of imaging studies

**Test**	**Number studies**	**Range LR+**	**Pooled LR+ (95% CI)**	**Range LR-**	**Pooled LR- (95% CI)**
**Localisation of infection**					

Ultrasound	20	1.6 to 55.0	3.5 (2.5, 4.8)	0.10 to 0.91	0.57 (0.47, 0.68)
Clinical features	5	1.1 to 26.6	-	0.09 to 0.89	-
Infection markers	10	1.0 to 8.8	-	0.09 to 1.00	-
Renal function markers	4	0.7 to 36.7	-	0.02 to 1.51	-
Immunofluorescence detection of bacteria	1	1.8	-	0.55	

**Detection of reflux**					

Ultrasound: standard	12	1.0 to 8.7	1.9 (1.2, 2.9)	0.05 to 0.98	0.76 (0.63, 0.93)
Ultrasound: contrast enhanced	16	3.8 to 71.2	14.1 (9.5, 20.8)	0.04 to 0.51	0.20 (0.13, 0.29)
Indirect radionuclide cystography	3	11.2 and 25.0	-	0.41 and 0.68	-

**Prediction of renal scarring**					

Ultrasound	2	1.3 to 3.0	-	0.60 to 0.86	-
Micturation cystourethrography	2	2.6 and 2.7	-	0.71 and 0.64	-
Temperature	1	1.1	-	0.44	-
CRP	1	1.1	-	0.44	-
Intravenous pyelography	1	12.9	-	0.88	-
Acute renal scintigraphy	1	3.1	-	0.54	-

**Detection of renal scarring**					

Ultrasound	7	1.3 to 35.9	-	0.14 to 0.99	
Intravenous pyelography	4	10 to 171.3	-	0.15 to 0.80	-
Indirect radionuclide cystography	2	2.1 to 12.6	-	0.15 to 0.75	-

### Clinical effectiveness

One RCT evaluated the effectiveness of routine follow-up investigation for children with confirmed UTI. This study was published as an abstract and we are unable to obtain further data[19].

The objective was to determine whether routine imaging, using ultrasound and MCUG, of children with their first UTI significantly reduced renal scarring or recurrent UTI. Children aged 2–10 years (n = 172), with confirmed UTI, were allocated to routine (all received Ultrasound and MCUG) or selected imaging (Ultrasound and MCUG for recurrent UTI or persistent problems). Routine investigation lead to higher rates of imaging (100% vs 21%), identification of VUR, and antibiotic prophylaxis compared to the selective investigation group. However, there was no difference in the proportion of children with recurrent UTI or in the rate of renal scarring between the two groups after two years of follow-up. The authors concluded that routine imaging of toilet trained pre-school and school aged children with their first uncomplicated UTI is not worthwhile.

### Diagnostic accuracy

None of the studies fulfilled all QUADAS criteria. Inadequate reporting was a problem in many studies; only two studies reported sufficient information to determine whether each criterion had been met. Less than half of studies included an appropriate spectrum of patients, and reported selection criteria. Incorporation bias, verification bias, and disease progression bias were also inadequately addressed by around half of all studies. Results of the quality assessment are presented [see Additional file 2].

#### Localisation of infection

**Figure 2:**
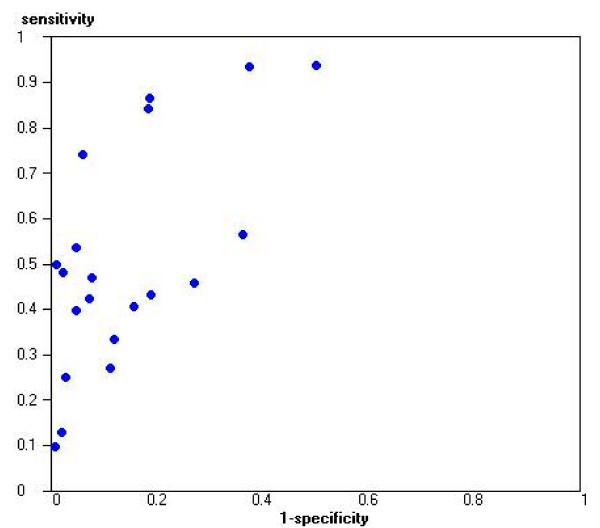
Sensitivity and specificity plotted in ROC space for ultrasound for the localisation of infection

A total of 31 studies (61 evaluations) evaluated the diagnostic accuracy of one or more tests to localise infection. Ultrasound was evaluated in 20 studies [20-39]. Performance was poor both in terms of ruling in and ruling out renal involvement: the pooled positive likelihood ratio was 3.5 (95% CI: 2.5, 4.8), and the pooled negative likelihood ratio was 0.57 (95% CI: 0.47, 0.68). Figure 2 shows estimates of sensitivity and specificity for these studies plotted in ROC space. There was significant between study heterogeneity in the ultrasound evaluations (p < 0.0001). None of the items investigated in the regression analysis showed a significant association with the DOR. Thirteen studies investigated clinical or laboratory-based tests[22,25,40-50]. The tests investigated varied greatly, and in general, showed poor performance.

#### Detection of VUR

**Figure 3:**
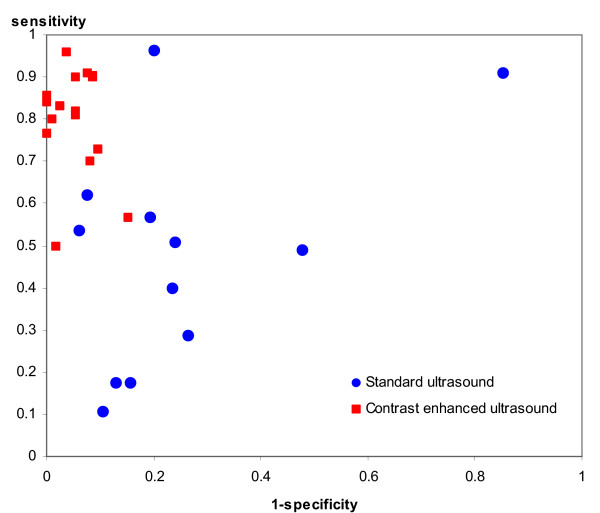
Sensitivity and specificity plotted in ROC space for ultrasound for the detection of reflux

We identified 30 studies (40 evaluations) evaluating the diagnostic accuracy of tests to detect VUR. Ultrasound was assessed in 28 studies, 12 using standard ultrasound techniques[35,51-61], and 16 using cystosonography or other contrast-enhanced techniques [62-77]. Standard ultrasound techniques performed poorly: the pooled positive LR was 1.9 (95% CI: 1.2, 2.9), and the pooled negative LR was 0.76 (95% CI: 0.63, 0.93) Contrast-enhanced ultrasound techniques showed much better performance, with higher pooled positive likelihood ratios (14.1, 95% CI: 9.5, 20.8) and lower pooled negative likelihood ratios (0.20, 95% CI: 0.13, 0.29). Figure 3 shows estimates of sensitivity and specificity for all ultrasound studies plotted in ROC space. Both techniques showed between study heterogeneity (p < 0.001). Regression analysis found that ultrasound technique, disease progression bias and use of an appropriate reference standard showed a significant association with the DOR. The DOR was 16.87 times greater (95% CI: 7.03, 40.48) in studies that used contrast enhanced ultrasound rather than standard ultrasound; 2.65 (95% CI: 1.02, 6.90) times higher in studies in which there was no clinically significant delay between the index test and reference standard; and 7.14 (95% CI: 1.13, 50) times higher in studies that used an inappropriate reference standard.

Two studies evaluated indirect radionuclide voiding cystography[78,79]. These reported good positive likelihood ratios (11.2 and 25.0), but negative likelihood ratios were poor (0.41 and 0.68).

#### Prediction of renal scarring

Five studies (nine evaluations) investigated the ability of a variety of tests (clinical, laboratory-based, and imaging techniques) to predict renal scarring[29,32,80,81]. The diagnostic accuracies reported in these studies were poor. Positive LRs were in the range of 1.1–3.1 for four of the studies, with the fifth (the only study of IVP) reporting a positive LR of 12.9. Negative likelihood ratios ranged from 0.44 and 0.88.

#### Detection of renal scarring

**Figure 4:**
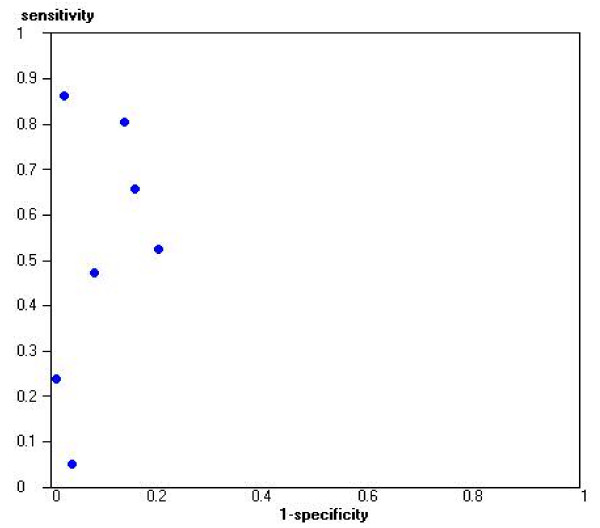
Sensitivity and specificity plotted in ROC space for ultrasound for the detection of scarring

Thirteen studies evaluated the diagnostic accuracy of tests to detect renal scarring. Four studies evaluated the diagnostic accuracy of IVP: positive likelihood ratios were high (10.0 to 171.3), but corresponding negative likelihood ratios were poor (0.15 to 0.80)[80,82-84]. Only one study included an appropriate patient spectrum[80], and this reported a much lower positive likelihood ratio (10.0 compared to next lowest of 58.8), and higher negative likelihood ratio (0.80 compared to next highest of 0.42) than the others. Seven studies evaluated standard ultrasound techniques[60,85-90]. Figure 4 shows estimates of sensitivity and specificity for these studies plotted in ROC space. Performance characteristics varied greatly, although positive likelihood ratios were generally moderate to high (1.3–35.9). Negative likelihood ratios showed poorer performance for ruling out scarring (0.14–0.99). Three studies assessed the diagnostic accuracy of indirect radionuclide cystography[78,84,91]; positive likelihood ratios were moderate and ranged from 3.3 to 12.6, and negative likelihood ratios ranged from 0.15 to 0.63.

## Discussion

When considering further testing following confirmation of UTI, it is important to bear in mind clinical aim. If the information derived cannot be used to prevent renal disease there is little benefit in testing. Tests should only be conducted if (a) the results will lead to a change in management and (b) this change is likely to lead to an improved outcome. For this reason, the ideal study would be a randomised controlled trial of different testing strategies, or no testing. We identified only one such study, and this was only available as an abstract reporting limited information, and we were unsuccessful in obtaining further details[19]. Our results are therefore primarily derived from diagnostic accuracy studies, which assume the validity of the clinical aims of current testing.

Localisation of infection can be considered a first step in the investigation of UTI. Lower UTI does not involve the kidneys and so cannot lead to renal scarring. Children with lower UTI are therefore unlikely to benefit from immediate investigation. Given that therapeutic delay is thought to be associated with renal damage[92], the possibility that they may benefit from monitoring for recurrence remains open to question. The ideal test to localise infection would be non-invasive, inexpensive, and quick to perform. Further investigation of children with infections of the lower urinary tract could thus be avoided. Our results do not support the use of any of the minimally invasive tests evaluated as alternatives to renal scintigraphy for the localisation of infection. However, the available evidence was limited, and further primary research in this area would be useful. Testing with the specific aim of localisation of infection is not common in current practice. Baseline renal scintigraphy in all children with confirmed UTI, in whom further investigation is planned, may be beneficial. This approach would eliminate a substantial proportion of children from further invasive investigations.

The detection of VUR has historically been considered an important element in the investigation of UTI, as it has been thought to indicate an increased risk of scarring. This idea is currently the subject of considerable debate. The only study of the effectiveness of imaging identified by our review compared routine and selective imaging, using US and MCUG for the detection of VUR[19]. This study found increased rates of VUR detection and prophylaxis with routine imaging, but no reduction in scarring or recurrent UTIs. Other studies have shown that the presence of VUR, as determined by MCUG, correlates poorly with the presence of renal scarring[78,93-95]. A recent systematic review also found that VUR is a weak predictor for renal damage in children hospitalised with UTI[96]. The management of VUR and how this impacts on the risk of future renal disease is also the subject of debate. A clinical trial comparing surgical and medical management found no difference in outcome[97], and a systematic review evaluating antimicrobial prophylaxis for the prevention of UTI in children found a lack of data for children with VUR[98]. Given the considerable doubts surrounding both the link between VUR and renal scarring, and the benefits to be derived from treating VUR, it appears difficult to justify the routine use of MCUG. This is an invasive, and costly test, involving considerable exposure to ionising radiation; its use should therefore be minimised where possible. Should the evaluation of VUR be considered clinically necessary, the available evidence supports the use of contrast-enhanced ultrasound techniques as an accurate alternative. Although not currently in widespread use in the UK, these techniques have the advantage of not involving exposure to ionising radiation. In addition, standard ultrasound forms part of the examination, allowing simultaneous screening for anatomical abnormalities and some types of calculi.

A test predicting risk of renal scarring would be useful were a treatment available to prevent its development. Anti-microbial therapy is usually initiated in children with confirmed UTI prior to investigation, as there is some evidence that treatment delay may affect scarring[3,7]. Predicting risk of renal scarring as a result of a current infection would, therefore, appear to be of academic interest alone. We identified no acute tests that were able to accurately predict the development of renal scarring. The prediction of risk of future infection is of potential interest in guiding the initiation of prophylactic antimicrobial therapy, but is outside the scope of this paper.

Although the presence of renal scarring represents the initial stages of renal disease, there is little that can be done to treat patients with scarring in order to prevent complications. If progressive scarring is assumed to be the consequence of repeat infections then anti-microbial prophylaxis may be initiated, although the effectiveness of this strategy remains open to debate. Imaging for the detection of renal scarring may be seen as a means of monitoring disease progression. If repeat examination is required then a less invasive alternative to the reference standard (renal scintigraphy), and one which avoids the use of ionising radiation, would seem particularly desirable. We found standard ultrasound examination to be a potentially useful test for ruling in scarring, but poor for ruling it out. This fits with anecdotal opinion that ultrasound is good at identifying gross scarring, but poor at detecting minor lesions. It may be that ultrasound images are insufficiently subtle to enable their use in monitoring disease progression. Further research on the accuracy of ultrasound in grading scarring is therefore required. Indirect radionuclide cystography is sometimes advocated as an alternative test for renal scarring, on the grounds that it may combine detection of VUR and scarring in a single test. We found it to be an accurate test for scarring, but poor for ruling out VUR.

## Conclusion

There is no evidence to support the clinical effectiveness of routine investigation of children with confirmed UTI. There is limited evidence that routine imaging to detect VUR, following first UTI in older children, has no effect on recurrence or renal scarring.

High quality primary research on the effectiveness, in terms of improved patient outcome, of testing at all stages in the investigation of confirmed UTI is urgently required.

## Competing interests

The author(s) declare that they have no competing interests.

## Authors' contributions

All authors contributed towards the conception and design of the study and the interpretation of the data. They also read and approved the final manuscript. MEW and PFW participated in data extraction, the analysis of data, and drafted the article.

## Pre-publication history

The pre-publication history for this paper can be accessed here:



## Supplementary Material

Additional File 1 Additional file 1 is a Microsoft Word file containing a table of the results of individual studies included in the review. Click here for file

Additional File 2 Additional file 2 is a Microsoft Word file containing a table of the results of the quality assessment of included studies. Click here for file
