# Supplementary material for: Further investigation of confirmed urinary tract infection (UTI) in children under five years: a systematic review
Source: BMC Pediatr. 2005 Mar 15;5:2. doi: 10.1186/1471-2431-5-2 (PMC1079875; doi:10.1186/1471-2431-5-2)
Supplement: Additional File 1 — is a Microsoft Word file containing a table of the results of individual studies included in the review. [file 1471-2431-5-2-S1.doc]

## Additional Table 1 Individual study results

| **Study details** | **Test details; time** | **Definition of positive result** | **Unit of analysis** | **a** | **b** | **c** | **d** | **Sens** | **Spec** | **DOR** | **LR+** | **LR-** |
| --- | --- | --- | --- | --- | --- | --- | --- | --- | --- | --- | --- | --- |
| **Localisation of infection** | | | | | | | | | | | | |
| **Ultrasound** | | | | | | | | | | | | |
| Andrich (1992)^16^ | Standard; not stated | Not stated | Patients | 3 | 0 | 23 | 24 | 11.5 | 100.0 | 7.3 | 6.5 | 0.88 |
| Benador (1994)^17^  (>= 1yr, any UTI) | Standard; acute | Renal changes indicative of APN | Patients | 12 | 3 | 19 | 6 | 38.7 | 66.7 | 1.2 | 1.1 | 0.94 |
| (>= 1yr, 1^st^ UTI) |  |  |  | 5 | 2 | 13 | 1 | 27.8 | 33.3 | 0.2 | 0.5 | 1.89 |
| (>= 1yr, multiple UTI) |  |  |  | 7 | 1 | 6 | 5 | 53.8 | 83.3 | 4.2 | 2.5 | 0.59 |
| (< 1yr, any UTI) |  |  |  | 20 | 4 | 23 | 24 | 46.5 | 85.7 | 4.7 | 3.0 | 0.63 |
| (< 1yr, 1^st^ UTI) |  |  |  | 18 | 4 | 22 | 22 | 45.0 | 84.6 | 4.1 | 2.7 | 0.66 |
| (< 1yr, multiple UTI) |  |  |  | 2 | 0 | 1 | 2 | 66.7 | 100.0 | 8.3 | 3.8 | 0.45 |
| (all ages, any UTI) |  |  |  | 32 | 7 | 42 | 30 | 43.2 | 81.1 | 3.1 | 2.3 | 0.70 |
| (all ages, 1^st^ UTI) |  |  |  | 23 | 6 | 35 | 23 | 39.7 | 79.3 | 2.4 | 1.8 | 0.77 |
| (all ages, multiple UTI) |  |  |  | 9 | 1 | 7 | 7 | 56.3 | 87.5 | 6.3 | 3.4 | 0.53 |
| Biggi (2001)^18^ | Standard; not stated | Renal changes indicative of APN | Renal units | 19 | 12 | 51 | 96 | 27.1 | 88.9 | 2.9 | 2.4 | 0.82 |
| Bircan (1995)^19^ | Standard; acute | Renal changes indicative of APN and presence of congenital abnormalities | Patients | 11 | 0 | 34 | 18 | 24.4 | 100.0 | 12.3 | 9.5 | 0.76 |
| Boudailliez (1998)^20^ | Doppler; not stated | Not stated | Renal units | 10 | 8 | 20 | 60 | 33.3 | 88.2 | 3.6 | 2.8 | 0.76 |
| Bykov (2003)^34^ | Doppler; acute | Renal changes indicative of APN | Renal units | 20 | 3 | 7 | 48 | 74.1 | 94.1 | 45.7 | 12.6 | 0.28 |
| Girona (1995)^21^ | Standard; not stated | Abnormal kidney size | Renal units | 17 | 36 | 20 | 97 | 45.9 | 72.9 | 2.3 | 1.7 | 0.74 |
| Guermazi (1993)^22^ | Standard; not stated | Renal changes indicative of APN or scarring | Patients | 14 | 5 | 19 | 64 | 42.4 | 92.8 | 8.7 | 5.9 | 0.62 |
| Hajjar (2002)^23^ | Doppler; acute | Renal changes indicative of APN | Patients | 15 | 1 | 13 | 20 | 53.6 | 95.2 | 15.7 | 11.3 | 0.49 |
| Hitzel (2002)^24^ | Doppler; acute | Renal changes indicative of APN | Patients | 44 | 5 | 3 | 5 | 93.6 | 50.0 | 12.7 | 1.9 | 0.13 |
|  |  |  | Renal units | 43 | 11 | 11 | 48 | 79.6 | 81.4 | 16.0 | 4.1 | 0.26 |
| Hitzel (2000)^25^ | Colour Doppler; not stated | Not stated | Renal units | 43 | 11 | 8 | 49 | 84.3 | 81.7 | 22.0 | 4.6 | 0.19 |
| Ilyas (2002)^26^ | Standard; acute | Renal changes indicative of APN | Patients | 9 | 0 | 89 | 65 | 9.2 | 100.0 | 13.9 | 12.7 | 0.91 |
| Jakobsson (1992)^27^ | Standard; acute | Renal changes indicative of APN | Renal units | 61 | 13 | 47 | 23 | 56.5 | 63.9 | 2.3 | 1.6 | 0.69 |
| Jequier (1998)^28^ | Standard; acute | Renal changes indicative of APN | Patients | 71 | 18 | 104 | 97 | 40.6 | 84.3 | 3.6 | 2.6 | 0.70 |
|  | Doppler; acute |  |  | 22 | 1 | 89 | 61 | 19.8 | 98.4 | 10.3 | 8.4 | 0.82 |
| Krzemien (2002)^29^ | Doppler; acute | Renal changes indicative of APN | Renal units | 15 | 2 | 17 | 24 | 46.9 | 92.3 | 8.7 | 6.1 | 0.58 |
| Lavocat (1997)^30^ | Standard; acute | Renal changes indicative of APN | Renal units | 28 | 0 | 28 | 54 | 50.0 | 100.0 | 109.0 | 55.0 | 0.50 |
| Morin (1999)^31^ | Standard; acute | Renal changes indicative of APN | Patients | 58 | 3 | 4 | 5 | 93.5 | 62.5 | 20.4 | 2.5 | 0.10 |
| Muro (2002)^35^ | Doppler; acute | Renal changes indicative of APN | Patients | 52 | 5 | 8 | 22 | 86.7 | 81.5 | 28.6 | 4.7 | 0.16 |
| Sfakianakis (1989)^32^ | Standard; not stated | Not stated | Patients | 12 | 0 | 13 | 23 | 48.0 | 100.0 | 43.5 | 23.1 | 0.52 |
| Sreenarasimhalah (1995)^33^ | Standard; acute | Not stated | Renal units | 21 | 2 | 32 | 41 | 39.6 | 95.3 | 11.0 | 8.5 | 0.63 |
| **Clinical features of acute pyelonephritis (APN)** | | | | | | | | | | | | |
| Biggi (2001)^18^ | Temperature; acute | >=39.1°C | Patients | 45 | 18 | 25 | 12 | 64.3 | 40.0 | 1.2 | 1.1 | 0.89 |
| Buyan (1993)^36^ | Flank pain, chills, nausea, vomiting, fever, tenderness of the costo-vertebral angle; acute | Presence of any symptoms | Patients | 12 | 0 | 9 | 3 | 57.1 | 100.0 | 9.2 | 4.5 | 0.49 |
| Everaert (1998)^37^ | Not stated; acute | Symptoms of APN | Patients | 31 | 0 | 13 | 18 | 70.5 | 100.0 | 86.3 | 26.6 | 0.31 |
| Fretzayas (2000)^38^ | Temperature; acute | >=38°C | Patients | 26 | 19 | 4 | 34 | 86.7 | 64.2 | 10.4 | 2.4 | 0.23 |
| Landau (1994)^39^ | Physical examination; blood WBC; band forms; UA; WBC in stool when diarrhoea present); acute | Presence of any symptoms | Patients | 48 | 53 | 1 | 26 | 98.0 | 32.9 | 16.0 | 1.5 | 0.09 |
| **Infection markers** | | | | | | | | | | | | |
| Biggi (2001)^18^ | C-reactive protein (CRP); acute | >880 mg/l | Patients | 45 | 10 | 25 | 21 | 64.3 | 67.7 | 3.7 | 2.0 | 0.53 |
| Buyan (1993)^36^ | C-reactive protein (CRP); acute | >20ug/l | Patients | 3 | 0 | 18 | 3 | 14.3 | 100.0 | 1.3 | 1.3 | 0.96 |
| Fretzayas (2000)^38^ | C-reactive protein (CRP); acute | >200 mg/l | Patients | 21 | 23 | 9 | 30 | 70.0 | 56.6 | 2.9 | 1.6 | 0.54 |
| Gervaix (2001)^40^ | C-reactive protein (CRP); acute | >=400 mg/l | Patients | 23 | 9 | 11 | 11 | 67.6 | 55.0 | 2.5 | 1.5 | 0.60 |
| Girona (1995)^21^ | C-reactive protein (CRP); acute | >20 mg/l | Patients | 18 | 14 | 3 | 21 | 85.7 | 60.0 | 7.8 | 2.1 | 0.27 |
| Prat (2003)^46^ | C-reactive protein (CRP); acute | >=20 mg/l | Patients | 12 | 42 | 1 | 22 | 92.3 | 34.3 | 6.3 | 1.4 | 0.22 |
| Smolkin (2002)^41^ | C-reactive protein (CRP); acute | >=20mg/l | Patients | 18 | 34 | 0 | 8 | 100.0 | 19.0 | 9.1 | 1.2 | 0.13 |
| Stokland (1996)^42^ | C-reactive protein (CRP); acute | >20mg/l | Patients | 69 | 73 | 4 | 29 | 94.5 | 28.4 | 6.2 | 1.3 | 0.21 |
| **Renal function markers** | | | | | | | | | | | | |
| Biggi (2001)^18^ | Erythrocyte sedimentation rate (ESR); acute | >68 mm/h | Patients | 34 | 15 | 36 | 16 | 48.6 | 51.6 | 1.0 | 1.0 | 1.00 |
| Buyan (1993)^36^ | Erythrocyte sedimentation rate (ESR); acute | >25 mm/h | Patients | 7 | 0 | 14 | 3 | 33.3 | 100.0 | 3.6 | 2.7 | 0.75 |
| Fretzayas (2000)^38^ | Erythrocyte sedimentation rate (ESR); acute | 30 mm/hour | Patients | 27 | 22 | 3 | 31 | 90.0 | 58.5 | 11.0 | 2.1 | 0.19 |
| Biggi (2001)^18^ | Microscopy; acute | >14 601 WBC/mm^3 | Patients | 39 | 13 | 31 | 18 | 55.7 | 58.1 | 1.7 | 1.3 | 0.77 |
|  |  | >52% granulocytes |  | 36 | 11 | 34 | 20 | 51.4 | 64.5 | 1.9 | 1.4 | 0.76 |
| Buyan (1993)^36^ | Microscopy; acute | >15 000 WBC/cm | Patients | 5 | 0 | 16 | 3 | 23.8 | 100.0 | 2.3 | 2.0 | 0.86 |
| Landau (1994)^43^ | Microscopy; acute | >=5 WBC/hpf | Patients | 48 | 56 | 4 | 34 | 92.3 | 37.8 | 6.6 | 1.5 | 0.22 |
| Landau (1994)^39^ | Microscopy; acute | >=5 WBC/hpf | Patients | 45 | 52 | 4 | 27 | 91.8 | 34.2 | 5.3 | 1.4 | 0.26 |
| Fretzayas (2000)^38^ | Polymorphonuclear elastase-α1-antitrypsin complex; acute | 95th percentile of reference range | Patients | 29 | 27 | 1 | 26 | 96.7 | 49.1 | 19.0 | 1.9 | 0.10 |
| Gervaix (2001)^40^ | Procalcitonin (PCT); acute | >=0.5ng/ml | Patients | 25 | 3 | 9 | 17 | 73.5 | 85.0 | 13.4 | 4.4 | 0.33 |
| Prat (2003)^46^ | Procalcitonin (PCT); acute | >=1.0ng/ml | Patients | 12 | 24 | 1 | 40 | 92.3 | 62.5 | 20.0 | 2.5 | 0.41 |
| Smolkin (2002)^41^ | Procalcitonin (PCT); acute | >=0.5ug/l | Patients | 17 | 4 | 1 | 38 | 94.4 | 90.5 | 99.8 | 8.8 | 0.09 |
| Jantausch (1994)^44^ | B_2_M; acute | >= 0.5 ug/mg CR | Patients | 10 | 2 | 4 | 1 | 71.4 | 33.3 | 1.4 | 1.1 | 0.80 |
|  | NAG/creatinine ratio and B_2_M; acute | Not stated | Patients | 6 | 2 | 8 | 1 | 42.9 | 33.3 | 0.5 | 0.7 | 1.51 |
| Capa Kaya (2001)^45^ | NAG and NAG/creatinine ratio; acute | 5 U/l for NAG, 7U/g for NAG creatinine ratio | Patients | 30 | 5 | 0 | 65 | 100.0 | 92.9 | 726.5 | 12.7 | 0.02 |
| Jantausch (1994)^44^ | NAG/creatinine ratio; acute | >= 40 umol/hour/mg CR | Patients | 13 | 4 | 6 | 1 | 68.4 | 20.0 | 0.7 | 0.9 | 1.30 |
| Everaert (1998)^37^ | Urinary alpha-1-MG-creatinine ratio; acute | >10mg/g | Patients | 43 | 0 | 1 | 18 | 97.7 | 100.0 | 1073 | 36.7 | 0.03 |
| **Immunofluorescence detection of antibody coated bacteria** | | | | | | | | | | | | |
| Buyan (1993)^36^ | Immunofluorescence; acute | >2 ACB/200 fields | Patients | 14 | 1 | 7 | 2 | 66.7 | 66.7 | 3.2 | 1.8 | 0.55 |
| **DETECTION OF REFLUX** | | | | | | | | | | | | |
| **Standard ultrasound** | | | | | | | | | | | | |
| Baronciani (1986)^47^ | Standard | Presence of reflux: dilation or hydronephrosis | Patients | 13 | 4 | 8 | 49 | 61.9 | 92.5 | 17.5 | 8.2 | 0.41 |
| Evans (1999)^48^ | Standard | Presence of reflux (change in pelvic diameter) | Renal units | 2 | 10 | 17 | 84 | 10.5 | 89.4 | 1.1 | 1.0 | 1.0 |
| Foresman (2001)^49^ | Duplex | Any abnormality | Patients | 24 | 43 | 25 | 47 | 49.0 | 52.2 | 1.0 | 1.0 | 0.98 |
| Mage (1989)^50^ | Standard | Not stated | Patients | 22 | 5 | 19 | 76 | 53.7 | 93.8 | 16.0 | 8.7 | 0.49 |
| Mahant (2002)^51^ | Standard | Presence of reflux (dilation) | Patients | 14 | 30 | 21 | 97 | 40.0 | 76.4 | 2.2 | 1.7 | 0.79 |
| Morin (1999)^31^ | Standard | Renal changes indicative of APN | Patients | 20 | 41 | 2 | 7 | 90.9 | 14.6 | 1.5 | 1.1 | 0.62 |
| Muensterer (2002)^52^ | Standard | Abnormal kidney size or dilation vs. reflux >= grade 3 | Renal units | 21 | 118 | 2 | 245 | 91.3 | 67.5 | 17.8 | 2.8 | 0.15 |
|  |  | Presence of reflux (dilation) vs. presence of reflux | Renal units | 35 | 76 | 34 | 241 | 50.7 | 76.0 | 3.2 | 2.1 | 0.65 |
|  |  | Abnormal kidney size vs. presence of reflux | Renal units | 20 | 28 | 49 | 289 | 29.0 | 91.2 | 4.2 | 3.3 | 0.78 |
|  |  | Abnormal kidney size vs. reflux >= grade 3 | Renal units | 11 | 37 | 12 | 326 | 47.8 | 89.8 | 8.0 | 4.7 | 0.58 |
|  |  | Presence of reflux (dilation) vs. reflux >= grade 3 | Renal units | 18 | 93 | 5 | 270 | 78.3 | 74.4 | 9.7 | 3.0 | 0.31 |
| Oostenbrink (2000)^53^ | Standard | Presence of reflux (at least mild dilatation) | Patients | 21 | 20 | 16 | 83 | 56.8 | 80.6 | 5.3 | 2.9 | 0.54 |
| Salih (1994)^54^ | Colour doppler | Presence of reflux (blue-coloured jet) | Renal units | 26 | 3 | 1 | 12 | 96.3 | 80.0 | 63.1 | 4.8 | 0.05 |
| Tan (1988)^55^ | Standard | Not stated | Patients | 3 | 6 | 14 | 32 | 17.6 | 84.2 | 1.2 | 1.1 | 0.98 |
| Trave (1997)^56^ | Standard | Not stated | Renal units | 3 | 4 | 14 | 27 | 17.6 | 87.1 | 1.5 | 1.4 | 0.95 |
| Verber (1988)^57^ | Standard | Presence of reflux or scarring | Renal units | 8 | 9 | 20 | 25 | 28.6 | 73.5 | 1.1 | 1.1 | 0.97 |
| **Contrast-enhanced ultrasound** | | | | | | | | | | | | |
| Alzen (1994)^58^ | Air contrast | Not stated | Renal units | 20 | 6 | 2 | 73 | 90.9 | 92.4 | 92.7 | 12.0 | 0.10 |
| Bergius (1989)^59^ | Cystosonography (Isopaque) | Presence of reflux >= grade 3 (air bubbles) | Renal units | 19 | 1 | 2 | 226 | 90.5 | 99.6 | 1178 | 134.7 | 0.11 |
|  |  | Presence of reflux >= grade 2 or air bubbles | Renal units | 56 | 2 | 14 | 176 | 80.0 | 98.9 | 275.1 | 71.2 | 0.20 |
| Berrocal (2001)^60^ | Cystosonography (SH U 508A) | Presence of reflux (micro-bubbles) | Renal units | 94 | 29 | 10 | 307 | 90.4 | 91.4 | 93.8 | 10.5 | 0.11 |
|  |  | Presence of reflux (micro-bubbles) | Patients | 67 | 16 | 9 | 124 | 88.2 | 88.6 | 53.6 | 7.5 | 0.14 |
| Frutos (2000)^61^ | Cystosonography (Levograf) | Presence of reflux (micro-bubbles) | Renal units | 63 | 19 | 7 | 204 | 90.0 | 91.5 | 88.8 | 10.6 | 0.11 |
| Haberlick (1997)^62^ | Colour Doppler Cystosonography | Presence of reflux (blue-coloured jet) | Renal units | 21 | 10 | 9 | 114 | 70.0 | 91.9 | 24.7 | 8.7 | 0.33 |
| Kessler (1982)^63^ | Cystosonography (Cysto-Conray) | Presence of reflux (micro-bubbles and/or dilation) | Renal units | 13 | 0 | 4 | 38 | 76.5 | 100.0 | 231.0 | 58.5 | 0.24 |
| McEwing (2002) ^72^ | Cystosonography (Levovist) | Presence of reflux >= grade 2 | Patients | 9 | 0 | 5 | 83 | 64.3 | 100.0 | 288.5 | 106.4 | 0.37 |
|  |  | Presence of reflux >= grade 2 | Renal units | 8 | 3 | 8 | 173 | 50.0 | 98.3 | 57.7 | 29.3 | 0.51 |
| Mentzel (2002)^64^ | Cystosonography (Levovist) | Presence of reflux | Renal units | 36 | 10 | 4 | 174 | 90.0 | 94.6 | 134.8 | 16.6 | 0.11 |
| Nakamura (2002)^71^ | Cystosonography (Levovist) | Presence of reflux | Renal units | 9 | 3 | 2 | 52 | 81.8 | 94.5 | 78.0 | 15.0 | 0.19 |
| Piaggio (2003)^65^ | Cystosonography (Levovist) | Not stated | Renal units | 42 | 35 | 32 | 196 | 56.8 | 84.8 | 7.2 | 3.8 | 0.51 |
| Radmayr (2002)^66^ | Doppler Cystosonography (galactose based contrast agent) | Presence of reflux (micro-bubbles) | Renal units | 71 | 5 | 3 | 129 | 95.9 | 96.3 | 481.0 | 25.7 | 0.04 |
| Rohden (1995)^67^ | Cystosonography (Echovist) | Not stated | Patients | 6 | 0 | 1 | 19 | 85.7 | 100.0 | 169.0 | 32.5 | 0.14 |
| Schneider (1984)^68^ | Cystosonography (Conray FL/air) | Presence of reflux (increased separation in the central renal echo complex) | Renal units | 34 | 18 | 5 | 162 | 87.2 | 90.0 | 55.1 | 8.4 | 0.15 |
|  |  | Presence of reflux | Renal units | 46 | 15 | 17 | 141 | 73.0 | 90.4 | 24.3 | 7.6 | 0.30 |
| Siamplis (1996)^69^ | Cystosonography (air) | Not stated | Renal units | 15 | 4 | 3 | 154 | 83.3 | 97.5 | 152.0 | 32.9 | 0.17 |
|  | Cystosonography (fluid) |  | Renal units | 17 | 8 | 1 | 150 | 94.4 | 94.9 | 206.6 | 17.2 | 0.08 |
| Uhl (2003)^73^ | Cystosonography (Levovist) | Presence of reflux (micro-bubbles) | Renal units | 16 | 0 | 3 | 28 | 84.2 | 100.0 | 268.7 | 47.9 | 0.18 |
| Valentini (2001)^70^ | Grey scale Cystosonography (Levovist) | Presence of reflux (micro-bubbles) | Renal units | 34 | 4 | 8 | 72 | 81.0 | 94.7 | 65.4 | 15.4 | 0.20 |
|  | Colour Doppler Cystosonography (Levovist) | Presence of reflux (colour signals) | Renal units | 42 | 5 | 0 | 71 | 100.0 | 93.4 | 1105 | 13.8 | 0.01 |
| **Indirect radionuclide cystography** | | | | | | | | | | | | |
| De Sadeleer (1994)^74^ | Indirect radionuclide voiding cystography (Tc-99m-MAG3) | Presence of reflux | Renal units | 14 | 0 | 29 | 37 | 32.6 | 100.0 | 36.9 | 25.0 | 0.68 |
| Hedman (1978)^75^ | Dynamic micturating scintigraphy (Tc-99m-DTPA) | Not stated | Renal units | 13 | 4 | 8 | 77 | 61.9 | 95.1 | 27.4 | 11.2 | 0.41 |
| **PREDICTION OF RENAL SCARRING** | | | | | | | | | | | | |
| **Ultrasound** | | | | | | | | | | | | |
| Hitzel (2000)^25^ | Colour Doppler; not stated | Not stated | Renal units | 15 | 21 | 8 | 31 | 65.2 | 59.6 | 2.7 | 1.6 | 0.60 |
| Jequier (1998)^28^ | Doppler; acute | Renal changes indicative of APN | Patients | 18 | 3 | 49 | 34 | 26.9 | 91.9 | 3.7 | 3.0 | 0.80 |
|  | Standard; acute |  |  | 43 | 23 | 58 | 46 | 42.6 | 66.7 | 1.5 | 1.3 | 0.86 |
| **MCUG** | | | | | | | | | | | | |
| Stokland (1998)^76^ | MCUG; not stated | Presence of reflux | Renal units | 26 | 38 | 39 | 209 | 40.0 | 84.6 | 3.7 | 2.6 | 0.71 |
| Stokland (1996)^77^ | MCUG; acute | Presence of reflux | Patients | 28 | 17 | 31 | 80 | 47.5 | 82.5 | 4.2 | 2.7 | 0.64 |
| **Other** | | | | | | | | | | | | |
| Stokland (1996)^77^ | Temperature; acute | >=38.5°C | Patients | 54 | 78 | 5 | 20 | 91.5 | 20.4 | 2.6 | 1.1 | 0.44 |
|  | CRP; acute | >20 mg/l |  | 54 | 78 | 5 | 20 | 91.5 | 20.4 | 2.6 | 1.1 | 0.44 |
| Stokland (1998)^76^ | IVP; acute | Presence of renal scarring | Renal units | 8 | 2 | 57 | 247 | 12.3 | 99.2 | 14.6 | 12.9 | 0.88 |
|  | Scintigraphy (Tc-99m-DMSA); acute |  |  | 36 | 44 | 29 | 205 | 55.4 | 82.3 | 5.7 | 3.1 | 0.54 |
| **DETECTION OF RENAL SCARRING** | | | | | | | | | | | | |
| **Ultrasound** | | | | | | | | | | | | |
| Barry (1998)^81^ | Standard; 1-3 months | Presence of renal scarring | Renal units | 147 | 11 | 23 | 467 | 86.5 | 97.7 | 255.2 | 35.9 | 0.14 |
| LeQuesne (1986)^82^ | Standard; not stated | Presence of renal scarring or signs of reflux | Renal units | 22 | 5 | 5 | 34 | 81.5 | 87.2 | 25.7 | 5.8 | 0.23 |
| MacKenzie (1994)^83^ | Standard; acute | Any abnormality | Patients | 32 | 10 | 29 | 41 | 52.5 | 80.4 | 4.4 | 2.6 | 0.60 |
| Moorthy (2004)^95^ | Standard; 3-6 months | Presence of diffuse renal scarring | Renal units | 93 | 60 | 104 | 673 | 47.2 | 91.8 | 10.0 | 5.8 | 0.58 |
|  |  | Presence of focal renal scarring | Renal units | 12 | 12 | 219 | 687 | 5.2 | 98.2 | 3.1 | 2.9 | 0.97 |
| Mucci (1994)^84^ | Standard; not stated | Not stated | Patients | 5 | 1 | 17 | 170 | 22.7 | 99.4 | 35.7 | 27.4 | 0.77 |
| Scherz (1994)^85^ | Standard; not stated | Presence of renal scarring | Patients (asymptomatic) | 3 | 1 | 0 | 22 | 100.0 | 95.7 | 105.0 | 14.0 | 0.13 |
|  |  |  | Patients (symptomatic) | 9 | 9 | 6 | 33 | 60.0 | 78.6 | 5.2 | 2.7 | 0.52 |
|  |  |  | Patients (all) | 12 | 10 | 6 | 55 | 66.7 | 84.6 | 10.2 | 4.1 | 0.41 |
| Trave (1997)^56^ | Standard; acute | Not stated | Renal units | 1 | 1 | 28 | 36 | 3.4 | 97.3 | 1.3 | 1.3 | 0.99 |
| **IVP** | | | | | | | | | | | | |
| McLorie (1980)^78^ | IVP (Diatrizoate meglumine and diatrizoate sodium); not stated | Presence of renal scarring | Renal units | 24 | 0 | 5 | 35 | 82.8 | 100.0 | 316.3 | 58.8 | 0.19 |
| Merrick (1980)^79^ | IVP; not stated | Not stated | Renal units | 47 | 0 | 8 | 100 | 85.5 | 100.0 | 1123 | 171.3 | 0.15 |
| Pickworth (1992)^80^ | IVP; not stated | Not stated | Patients | 13 | 0 | 9 | 62 | 59.1 | 100.0 | 177.6 | 74.0 | 0.42 |
| Stokland (1998)^76^ | IVP; follow-up | Presence of renal scarring | Renal units | 14 | 5 | 51 | 244 | 21.5 | 98.0 | 12.5 | 10.0 | 0.80 |
| **Indirect radionuclide cystography** | | | | | | | | | | | | |
| Gordon (1992)^87^ | Dynamic including micturating (Tc-99m-MAG3); follow-up | Not stated | Renal units | 44 | 7 | 6 | 53 | 88.0 | 88.3 | 48.8 | 7.1 | 0.15 |
| Pickworth (1992)^80^ | Dynamic including micturating (Tc-99m-MAG3); not stated | Presence of renal scarring or reflux | Patients | 14 | 2 | 3 | 36 | 82.4 | 94.7 | 60.5 | 12.6 | 0.21 |
|  |  |  | Renal units | 34 | 38 | 54 | 174 | 38.6 | 82.1 | 2.9 | 2.1 | 0.75 |
| De Sadeleer (1994)^74^ | Indirect voiding radionuclide cystography (Tc-99m-MAG3); acute | Presence of reflux | Renal units | 12 | 5 | 14 | 33 | 46.2 | 86.8 | 5.3 | 3.3 | 0.63 |
