# Supplementary material for: Further investigation of confirmed urinary tract infection (UTI) in children under five years: a systematic review
Source: BMC Pediatr. 2005 Mar 15;5:2. doi: 10.1186/1471-2431-5-2 (PMC1079875; doi:10.1186/1471-2431-5-2)
Supplement: Additional File 2 — is a Microsoft Word file containing a table of the results of the quality assessment of included studies. [file 1471-2431-5-2-S2.doc]

## Additional Table 2. Results of quality assessment

| **Study details** | **Spectrum composition** | **Selection criteria** | **Disease progression bias** | **Partial verification bias** | **Differential verification bias** | **Incorporation bias** | **Test execution details** | **Reference execution details** | **Test review bias** | **Diagnostic review bias** | **Clinical review bias** | **Uninterpretable results** | **Withdrawals** |
| --- | --- | --- | --- | --- | --- | --- | --- | --- | --- | --- | --- | --- | --- |
| Alzen(1994)^58^ | - | - | + | + | + | + | - | - | ? | ? | ? | ? | ? |
| Andrich(1992)^16^ | - | - | ? | + | + | + | - | - | ? | ? | + | ? | - |
| Baronciani(1986)^47^ | + | - | ? | + | + | + | + | - | + | + | + | ? | - |
| Barry(1998)^81^ | ? | - | + | + | + | + | + | + | + | + | ? | + | + |
| Benador(1994)^17^ | - | + | + | + | + | + | + | + | + | + | ? | ? | ? |
| Bergius(1989)^59^ | - | - | + | + | + | + | + | - | ? | ? | ? | ? | ? |
| Berrocal(2001)^60^ | - | + | + | + | + | + | + | + | + | + | ? | + | + |
| Biggi(2001)^18^ | + | + | ? | + | + | + | - | + | + | ? | ? | ? | - |
| Bircan(1995)^19^ | + | - | + | + | + | + | - | + | + | + | ? | + | + |
| Boudailliez(1998)^20^ | - | + | + | + | + | + | - | - | ? | ? | ? | ? | ? |
| Buyan(1993)^36^ | + | + | + | + | + | + | + | + | ? | ? | ? | ? | ? |
| Bykov(2003)^34^ | + | - | + | + | + | + | + | + | + | + | ? | + | + |
| Capa Kaya(2001)^45^ | + | - | + | + | + | + | + | + | + | + | ? | ? | ? |
| De Sadeleer(1994)^74^ | - | - | + | + | + | + | + | + | + | ? | + | ? | ? |
| Evans(1999)^48^ | - | + | + | + | + | + | + | - | ? | ? | ? | ? | ? |
| Everaert(1998)^37^ | - | + | ? | + | + | + | + | + | ? | ? | ? | + | ? |
| Foresman(2001)^49^ | - | + | ? | - | + | + | + | - | + | ? | ? | ? | - |
| Fretzayas(2000)^38^ | + | + | + | + | + | + | - | + | ? | + | ? | ? | ? |
| Frutos(2000)^61^ | - | - | + | + | + | + | + | + | + | ? | ? | + | + |
| Gervaix(2001)^40^ | + | + | + | + | + | + | + | + | + | + | ? | + | ? |
| Girona(1995)^21^ | + | + | ? | + | + | + | - | + | ? | ? | ? | ? | - |
| Gordon(1992)^87^ | + | - | ? | + | + | + | + | + | + | + | ? | + | + |
| Guermazi(1993)^22^ | - | - | + | + | + | + | - | - | ? | ? | ? | ? | - |
| Haberlick(1997)^62^ | - | + | + | + | + | + | + | - | + | + | ? | ? | + |
| Hajjar(2002)^23^ | - | + | + | + | + | + | + | + | ? | ? | ? | ? | ? |
| Hedman(1978)^75^ | + | - | + | + | + | + | - | - | ? | ? | ? | ? | ? |
| Hitzel(2002)^24^ | - | + | ? | + | + | + | + | + | + | + | ? | ? | + |
| Hitzel(2000)^25^ | - | + | ? | + | + | + | - | - | + | + | ? | ? | + |
| Ilyas(2002)^26^ | - | + | + | + | + | + | - | + | ? | ? | ? | + | + |
| Jakobsson(1992)^27^ | - | + | + | + | + | + | + | - | ? | ? | ? | ? | - |
| Jantausch(1994)^44^ | + | + | ? | + | + | + | + | - | ? | ? | ? | + | + |
| Jequier(1998)^28^ | + | + | + | + | + | + | + | + | + | + | ? | + | + |
| Kessler(1982)^63^ | - | - | ? | + | + | + | + | - | + | + | ? | ? | ? |
| Krzemien(2002)^29^ | + | + | + | + | + | + | - | - | ? | ? | ? | ? | ? |
| Landau(1994)^39^ | - | + | ? | + | + | + | + | + | ? | ? | + | ? | + |
| Landau(1994)^43^ | - | - | ? | + | + | + | - | - | ? | ? | ? | ? | ? |
| Lavocat(1997)^30^ | + | + | + | + | + | + | + | + | ? | ? | ? | ? | + |
| LeQuesne(1986)^82^ | - | - | ? | + | + | + | - | - | + | + | ? | ? | ? |
| MacKenzie(1994)^83^ | + | + | + | + | + | + | + | + | ? | ? | ? | ? | - |
| Mage(1989)^50^ | + | + | + | + | + | + | - | - | + | ? | ? | + | ? |
| Mahant(2002)^51^ | - | + | ? | + | + | + | + | + | ? | ? | + | ? | + |
| McEwing(2002)^72^ | - | - | + | + | + | + | + | + | + | + | ? | + | + |
| McLorie(1980)^78^ | - | + | + | + | + | + | + | + | + | + | - | ? | ? |
| Mentzel(2002)^64^ | - | + | + | + | + | + | + | + | + | + | ? | + | + |
| Merrick(1980)^79^ | - | - | + | + | + | + | - | - | + | + | + | ? | ? |
| Moorthy(2004)^95^ | + | - | + | + | + | + | + | + | ? | ? | ? | ? | ? |
| Morin(1999)^31^ | - | + | + | + | + | + | + | + | + | + | ? | ? | ? |
| Mucci(1994)^84^ | - | - | ? | + | + | + | - | - | ? | ? | ? | ? | ? |
| Muensterer(2002)^52^ | - | + | + | + | + | + | + | - | ? | ? | ? | + | ? |
| Muro(2002)^35^ | + | - | + | + | + | + | + | - | ? | ? | ? | ? | - |
| Nakamura(2002)^71^ | - | - | + | + | + | + | + | + | - | - | ? | ? | ? |
| Oostenbrink(2000)^53^ | + | + | ? | + | + | + | - | - | ? | ? | ? | ? | ? |
| Piaggio(2003)^65^ | - | + | ? | + | + | + | + | - | ? | ? | ? | + | ? |
| Pickworth(1992)^80^ | - | - | - | ? | + | + | - | - | ? | ? | + | ? | - |
| Prat(2003)^46^ | + | - | + | + | + | + | + | - | ? | ? | ? | ? | ? |
| Radmayr(2002)^66^ | - | + | + | + | + | + | + | - | + | + | ? | + | + |
| Rohden(1995)^67^ | - | - | ? | + | + | + | + | - | ? | ? | ? | ? | ? |
| Salih(1994)^54^ | - | + | + | + | + | + | + | - | + | + | ? | ? | ? |
| Scherz(1994)^85^ | - | + | ? | + | + | + | + | + | ? | ? | ? | ? | - |
| Schneider(1984)^68^ | - | + | + | + | + | + | + | - | ? | ? | ? | ? | ? |
| Sfakianakis(1989)^32^ | + | - | ? | + | + | + | - | - | ? | ? | ? | ? | - |
| Siamplis(1996)^69^ | - | + | + | + | + | + | + | - | ? | ? | ? | ? | + |
| Smolkin(2002)^41^ | + | + | + | + | + | + | - | - | + | ? | ? | + | + |
| Sreenarasimhalah(1995)^33^ | + | + | + | + | + | + | - | + | ? | ? | ? | ? | - |
| Stokland(1996)^77^ | + | + | - | + | + | + | - | + | + | + | + | + | - |
| Stokland(1996)^42^ | + | + | + | + | + | + | - | + | + | + | ? | + | + |
| Stokland(1998)^76^ | + | + | - | + | + | + | + for DMSA - for others | + | + | + | ? | + | - |
| Tan(1988)^55^ | + | + | ? | - | ? | + | + | - | + | ? | ? | ? | - |
| Trave(1997)^56^ | + | - | + | + | + | + | - | + for DMSA - for MCUG | ? | ? | ? | ? | - |
| Uhl(2003)^73^ | - | - | + | + | + | + | + | + | - | - | ? | ? | ? |
| Valentini(2001)^70^ | - | + | + | + | + | + | + | + | + | + | ? | + | + |
| Verber(1988)^57^ | + | - | ? | - | + | + | + | + | ? | ? | ? | ? | - |
